# The Supply of Probationers

**Published:** 1907-05-04

**Authors:** 


### Nursing Administration.

### THE SUPPLY OF PROBATIONERS.

In many quarters throughout the country a change is observable in applicants for training as probationers; and chiefly it is becoming more and more difficult for matrons to find candidates, suitable in other respects, who are willing or able to pay a premium. Very curious variations exist with regard to premiums in different parts of the kingdom. In London, for instance, a premium is required in only one General Hospital containing over 100 beds, and this is the London Temperance Hospital. All others take their ordinary probationers without fee, although at ten of the London General Hospitals arrangements are in force for receiving paying probationers or pupils. In the Provinces the premiums required from intending probationers vary from £25 at the Bristol Royal Infirmary and the Bristol General Hospital to a mere entrance fee of a guinea at the Royal Portsmouth Hospital, the Salop Infirmary, and the Royal South Hants Infirmary. Excluding the mere entrance fees there are some seventeen Provincial hospitals which require premiums from their ordinary probationers. No Scottish hospital requires any premium from its probationers, but, oddly enough, all the Irish General Hospitals require a substantial sum down, ranging from £52 10s. at the Meath Hospital to £10 at the Limerick County Hospital and the Dublin House of Industry Hospitals.

Inquiry has shown that among the hospitals which impose a premium on probationers not a few have allowed the custom to fall into abeyance. It is found in effect not to result in the selection of the most desirable candidates. Indeed one matron of an exceptionally good Provincial Training School, which in times past could count on a never-failing supply of paying candidates, has stated that the limitation of probationers to those who can afford to pay results in the present day in being compelled to fall back on women who have failed to secure an entrance, without payment, into reputable hospitals. The same falling-off in candidates willing to pay for their training is felt in most of the training schools which make provision for "Special Probationers" or Lady Pupils. In one respect this is all for the good. The women who were nervous in old days about trusting themselves to the rigours of ordinary training, or whose parents dreaded their being associated with people "beneath them," have learned more common sense, and now dread nothing so much as being marked out as "beneath" the rest, because they are incapable of going through the ordinary course. The food and general accommodation in the modern training school have been levelled up, till they far surpass what was available for the most expensive paying probationer in days gone by. It is the glory of the nursing profession that it is the most democratic of openings for women, and that rich and poor meet on a common basis of knowledge and traditions in the sick-room. Hence the disappearance of artificial distinctions in the training school is all for the good.

Whether other causes are at work to account for the shortage of probationers willing to pay premiums is a more doubtful matter. One circumstance certainly seems to exercise an adverse influence in securing paying probationers, and this is unavoidable. We refer to the age at which probationers are received in General Hospitals. It is becoming more and more usual for girls of the upper midle classes, even where parents are able to support them, to aim at a career for themselves. But the five years which must elapse between the time when they leave school and the time when they can be received as probationers is frequently a bar to their taking up nursing. Supposing they resolve to wait until they are old enough, and then find that they fail for reasons of health or temperament, they are ever after at a disadvantage with regard to their future, for almost every other calling demands an early entrance. Moreover most parents will make an effort to part with a premium for putting their girls into the way of earning their living after leaving school, but when they have lived at home for some years, or desire to break off from some other kind of work which is bringing in money, the demand comes as something of a shock, and there is often great reluctance to encourage the idea. Thus it may be feared that many women who might make into excellent nurses get drawn away into other occupations owing to the necessary delay before they can commence their training.

There is, however, in most hospitals such an overwhelming number of candidates that it may be thought superfluous to comment on this fact. Superintendents of popular training schools may still often sigh for a short way with applicants such as used to be practised by a well-known matron, which consisted of measuring them in their stocking feet, and rejecting all those above or below the required standard. The influence upon the candidate of the years spent after she leaves school is incontestably great. It would be impossible to fix on any special calling which could be regarded as the best preparation for the work of the nurse in the future. But on one point all superintendents are agreed, and that is the extreme unsuitability of domestic service under modern conditions as a way of filling in these preparatory years. The combination of qualities which go to make up the good probationer is so rare that it is of the utmost importance that the range of choice should be as little restricted as possible. For that reason we welcome the disposition of managers not to insist upon premiums, and this especially in view of the peculiar conditions of the nurse's training which render it her second start in life, and often involve on her part a considerable pecuniary sacrifice.

